# The deterioration of starch physiochemical and minerals in high-quality indica rice under low-temperature stress during grain filling

**DOI:** 10.3389/fpls.2023.1295003

**Published:** 2024-01-22

**Authors:** Juan Yang, Xinzheng Zhang, De Wang, Jinshui Wu, Hang Xu, Yang Xiao, Hongjun Xie, Wanju Shi

**Affiliations:** ^1^ College of Agronomy, Hunan Agricultural University, Changsha, Hunan, China; ^2^ Hunan Rice Research Institute, Hunan Academy of Agricultural Sciences, Changsha, Hunan, China

**Keywords:** rice, grain quality, low temperature, mineral nutrients, starch

## Abstract

Low temperatures during the grain-filling phase have a detrimental effect on both the yield and quality of rice grains. However, the specific repercussions of low temperatures during this critical growth stage on grain quality and mineral nutrient composition in high-quality hybrid indica rice varieties have remained largely unexplored. The present study address this knowledge gap by subjecting eight high-quality indica rice varieties to two distinct temperature regimes: low temperature (19°C/15°C, day/night) and control temperature (28°C/22°C) during their grain-filling phase, and a comprehensive analysis of various quality traits, with a particular focus on mineral nutrients and their interrelationships were explored. Exposure of rice plants to low temperatures during early grain filling significantly impacts the physicochemical and nutritional properties. Specifically, low temperature increases the chalkiness rate and chalkiness degree, while decreases starch and amylopectin content, with varying effects on amylose, protein, and gelatinization temperature among rice varieties. Furthermore, crucial parameters like gelatinization enthalpy (ΔH), gelatinization temperature range (R), and peak height index (PHI) all significantly declined in response to low temperature. These detrimental effects extend to rice flour pasting properties, resulting in reduced breakdown, peak, trough, and final viscosities, along with increased setback. Notably, low temperature also had a significant impact on the mineral nutrient contents of brown rice, although the extent of this impact varied among different elements and rice varieties. A positive correlation is observed between brown rice mineral nutrient content and factors such as chalkiness, gelatinization temperature, peak viscosity, and breakdown, while a negative correlation is established with amylose content and setback. Moreover, positive correlations emerge among the mineral nutrient contents themselves, and these relationships are further accentuated in the context of low-temperature conditions. Therefore, enhancing mineral nutrient content and increasing rice plant resistance to chilling stress should be the focus of breeding efforts to improve rice quality.

## 1 Introduction

Rice (Oryza sativa L.) is a vital staple food that provides essential nutrients, including starch, protein, and various macro-micro nutrients, to more than half of the global population (Huang et al., 2016). With the world’s population on the rise and increasing economic development, there has been an improvement in the yield potential and grain qualities of rice (Peng et al., 2009). Moreover, the nutritional quality of rice has garnered increasing attention in recent years due to its crucial role in human health, particularly in regions such as Asia, Latin America, and parts of Africa, where rice provides up to 76% of the caloric intake (Fitzgerald et al., 2009; Huang et al., 2016). The macro-micronutrients in rice are not only essential components of the human body, but also cofactors in many important biological processes (Huang et al., 2020). Inadequate consumption of macro-micro nutrients from food can have severe health consequences, especially among children and women in developing countries (Du et al., 2013). Zinc deficiency can lead to anorexia, stunted growth, and underdeveloped intelligence (Xi et al., 2016), while iron deficiency causes anemia in over two billion people globally (Zhang et al., 2020). Lack of calcium is the main cause of osteoporosis, and manganese deficiency can result in developmental and growth defects (Chen et al., 2005). Therefore, enhancing the nutrition of rice could be of significant benefit to human health, especially in developing countries.

Double-season rice cropping is a prevalent rice production system in central China and other parts of Asia, it involves sequential cultivation of early and late-season rice from March to November, leading to higher rice production per unit land area than single-season rice systems (Peng et al., 2009). Although late-season rice typically exhibits superior grain quality than early-season rice (Dong et al., 2017), its production is often impacted by lower temperatures from mid-September, leading to reduced grain yield and poor quality (Zeng et al., 2017; Zhang et al., 2017). Low temperatures have caused significant losses in rice production, with losses of up to 20% of total production reported in the last century (Xiao and Song, 2011). The southern regions of the mid-lower reaches of the Yangtze River have seen a significant increase in the area exposed to low temperatures over the 2010s (Wang et al., 2019). Moreover, addressing the gap in knowledge surrounding low-temperature stress and its impact on rice is also crucial, particularly in temperate and high-altitude areas in the tropics, where it is a significant factor affecting the growth and development of rice (Razafindrazaka et al., 2020).

Rice quality traits include milling recovery, physical appearance, cooking and eating qualities, and nutritional value. Starch, accounting for around 90% of milled rice weight, receives high attention for its physicochemical properties (Hu et al., 2020). The synthesis and accumulation of starch were blocked leading to the deterioration of cooking and eating quality for rice, especially under abiotic stress (Ahmed et al., 2015; Siddik et al., 2019; Wada et al., 2019). Previous studies have reported that low temperatures during the grain-filling stage increased amylose content (Zhu et al., 2017), improved the short chain of amylopectin (Chun et al., 2015), and decreased the amylopectin content and relative crystallinity in starch (Hu et al., 2020; Ai et al., 2023). Therefore, low temperatures could decrease starch physicochemical properties, and consequently deteriorate the cooking and eating quality of rice. Zhu et al. (2017) found that low temperatures increased protein content and gelatinization temperature while decreasing total starch content in Japonica rice. However, Hu et al. (2020) showed that the effect of protein and total starch content was not significant under low temperatures, but the gelatinization temperature decreased significantly. Additionally, some studies reported that the amylose and protein content decreased under low temperatures (Chun et al., 2015; Ai et al., 2023). Most previous studies have focused on the effect of low temperature on rice quality traits in japonica rice (Oryza sativa L. subsp. japonica) (Chun et al., 2015; Zhu et al., 2017; Hu et al., 2020) or inbred indica (Oryza sativa L. subsp. indica) (Lu et al., 2022). However, research on high-quality hybrid indica varieties has been limited, even though they have been bred to meet the demand for good eating and cooking quality in recent years (Zeng et al., 2022). Furthermore, the differences in quality characteristics, such as amylose content and physicochemical properties, between India and japonica rice are well-recognized (Feng et al., 2017). And comparative analyses of grain quality in response to high temperatures during the grain-filling stage between indica and japonica have been conducted (Fan et al., 2023). Therefore, it is crucial to investigate the effect of low temperature on high-quality indica varieties, which plays an essential role in maintaining food security and has been highly demanded by consumers in recent years.

While there has been extensive research on the effect of temperature on starch properties of rice, less attention has been paid to the impact of lower temperatures on mineral nutrients. The accumulation of mineral elements in rice grains is not only influenced by genetic factors but also constrained by external environmental conditions (Du et al., 2013; Huang et al., 2016). For instance, increasing atmospheric CO_2_ or temperature can reduce the nutrient content, such as Zn and Fe, in wheat or rice (Myers et al., 2014; Chaturvedi et al., 2017). Moreover, the application of nitrogen fertilizer and water management can also affect the micro nutrient composition of grains. Gu et al. (2015) reported that increasing nitrogen fertilizer resulted in decreased levels of micro-nutrients such as Cu, Mg, and S, while Fe, Mn, Zn, Na, etc., increased. In contrast, Wang et al. (2018) found that a moderate level of nitrogen application was favorable for promoting the accumulation of micro-nutrients like Cu, Fe, Mn, and Zn in brown rice. Additionally, Xu et al. (2019) discovered that alternating wetting and moderate soil drying irrigation decreased the content of Cu, Fe, Mn, Mo, Se, and Zn in the brown rice. However, the effect of low temperature on the mineral nutrient composition of rice grains remains unclear. Furthermore, a complex correlation has been documented between within-grain minerals and minerals with different quality traits such as amylose content, protein content, gel consistency, and gelatinization temperature in rice (Jiang et al., 2007; Huang et al., 2016; Xi et al., 2016). However, the relationships between quality traits and mineral nutrients have not been explored under low-temperature conditions.

This study compares the grain quality traits and mineral nutrient contents of eight high-quality indica rice cultivars subjected to either low-temperature stress or a control condition during the grain filling stage, which is the most critical for rice quality in response to extreme temperatures. Therefore, the primary objectives of the studies were to investigate (i) the impact of lower temperature on grain quality, and mineral nutrients in high-quality indica rice, and (ii) the association between grain quality traits and mineral nutrients exposed to lower temperature conditions.

## 2 Materials and methods

### 2.1 Plant materials

Eight high-quality indica varieties, Taoyouxiangzhan (TYXZ), Yliangyou911 (YLY911), Yuzhenxiang (YZX), Taiyou390 (TY390), Huanghuazhan (HHZ), Jinliangyouhuazhan (JLYHZ), Longjingyou 534 (LJY534), and Nongxiang 42 (NX42) were selected in this study. The experiment was conducted in the research farm at Hunan Agricultural University, Changsha city (14°C11′N, 121°C15′E, 21 m asl), Hunan Province, China. Twenty days of seedlings were manually transplanted into plastic pots (28cm and 32cm in internal diameter and height, respectively) to grow. Plants were given a basal dressing of 5g pot^-1^ [commercial fertilizer, 20-10-15 (N-P_2_O_5_-K_2_O)] before transplanting. Water, weeds, pests, and diseases were completely controlled as requirements of local high-yield cultivation.

### 2.2 Temperature treatments and samplings

During the heading stage of the rice plants, two to three primary tillers which had headed on the same day from each plant were labeled. As previous research has shown that the second week of post-heading is the most critical period for rice quality in response to extreme temperatures (Siddik et al., 2019), a walk-in climate chamber-treated experiment was conducted to impose the temperature treatment at this period. Specifically, on the seventh day after marking, thirty plants were randomly selected and moved into two independent temperature-controlled growth rooms (2.6 m × 2.2m × 2.0 m in length, width, and height, respectively). The plants were subjected to two different temperature regimes for six consecutive days, a lower temperature of 19°C during the daytime (07:00 a.m. to 6:59 p.m.) and 15°C during nighttime (07:00 p.m. to 6:59 a.m.), and a control temperature of 28°C during the daytime and 22°C during nighttime. To monitor the temperature in the rice canopy, two stand-alone sensors (HOBO, MX2301A, USA) were placed in each growth room to measure the temperature at 10-minute intervals. Once the temperature treatments were completed, all plants were transferred outdoors. After physiological maturation, all the marked panicles on the individual plants for each variety and temperature treatment were collected. The panicles were manually threshed and then stored at room temperature for three months before determining the grain quality traits.

### 2.3 Determination of grain chalkiness

Three samples consisting of 200 grains each were randomly selected for each treatment, which was then dehulled. Grain chalkiness rate (%) and chalkiness degree were measured by a flatbed scanner (ScanMaker i800plus, MICROTEK, China) and analyzed with SC-E software (Hangzhou, Wanshen Detection Technology Co., Ltd., Hangzhou, China).

### 2.4 Total starch content, amylose, and amylopectin content

The total starch content was determined using the total starch kit (Suzhou Comin Biotechnology Co., Ltd, Suzhou, China) in accordance with the kit’s protocol. The amylose content was measured using the amylose–iodine reaction with reference to the national standards of the People’s Republic of China (GB/T 17891-2017). To measure the amylose content, rice flour (100mg) was mixed with 1 ml of 95% ethanol and 9 mL of 1 M NaOH, and then boiled for 10 min. After cooling, the volume was made up to 100 ml with distilled water. 5 ml of solution was added with 1 ml of 1M aqueous acetic acid and 2 ml of iodine solution (0.2 g iodine and 2.0 g potassium iodide in 100 ml aqueous solution). The volume was then made up to 100ml with distilled water and the absorbance of the solution was measured at 620 nm with a spectrophotometer. The amylopectin content was obtained by subtracting the amylose content from the total starch content, as described previously by Zhu et al. (2021).

### 2.5 Determination of protein content

Total protein content was measured using the method by Hu et al. (2020) with some modifications. In brief, it was determined indirectly using nitrogen concentration estimated by the semi-microKjeldahl method and a Kjeldahl conversion coefficient of 5.95 was used with reference to the national standards of the People’s Republic of China (GB/T 5009.5-2016).

### 2.6 Determination of thermal properties

The thermal properties were determined using a differential scanning calorimetry analyzer (DSC 25, TA Instruments, USA). Five milligrams of starch were mixed with 10ul water, and then the mixture was hermetically sealed and left to stand at room temperature for 24 hours before being heated in the DSC. The DSC analyzer was calibrated using an empty aluminum pan as a reference. The sample pans were heated from 30°C to 95°C at a rate of 10°C min^-1^. Onset temperature (To), peak temperature (Tp), conclusion temperature (Tc), and gelatinization enthalpy (ΔH) were calculated by the TA Universal Analysis 2000 software. The gelatinization temperature range (R) and peak height index (PHI) were calculated as R = Tc−To and PHI =ΔH/(Tp− To) respectively (Ai et al., 2023).

### 2.7 Determination of pasting properties

The pasting properties of rice flour were evaluated using a rapid viscosity analyzer (RVA-3D, Newport Scientific, Australia). Specifically, 3 grams of rice flour were accurately weighed and placed into an RVA sample canister. Next, 25ml of ultrapure water was added to the canister, after which it was transferred into the RVA for testing. The temperature within the RVA tank was subjected to a heating-cooling program that commenced at 50°C for one minute, followed by a gradual increase to 95°C at a rate of 12°C per minute. The temperature was then maintained at 95°C for 2.5 minutes, after which it was decreased to 50°C at a rate of 12°C min^-1^ and maintained at 50°C for 2 minutes. The peak viscosity (PV), trough viscosity (TV), final viscosity (FV), breakdown value (PV - TV) and setback value (FV - PV) were analyzed by TCW (Thermal Cline for Windows) program (Jiang et al., 2022).

### 2.8 Macro and micro nutrients

The nutrients (B, Na, Mg, P, K, Ca, Mn, Fe, Cu, and Zn) were determined via an inductively coupled plasma mass spectrometer (ICP-MS; NexION300X; PerkinElmer, USA). Milled grains (0.50 g) were finely ground and wet-digested in a 50 ml conical flask using 10 ml mixed acid (4:1 HNO_3_–HClO_4_). After cooling, the digested solution was transferred to a 100 ml volumetric flask and brought to a final volume of 100 ml with double distilled water. The resulting solution was then filtered using a 0.45 um filter and collected in a fresh 10 ml plastic centrifuge tube for subsequent analysis by ICP-MS. Calibration curves and element contents were obtained using standard solutions of 32 elements (GNM-M323115-2013, 20DC821, China).

### 2.9 Statistical analysis

The statistical analysis employed in this study involved factorial analysis of variance (ANOVA), which was performed using the R language (version 4.1.0; http://www.R-project.org). Mean values ± standard error derived from three replications were reported in all tables and figures. To conduct multiple comparisons of variables with statistically significant differences (*p* < 0.05), Duncan’s new multiple range method was utilized. The figures were plotted using the Sigma Plot software version 14.0 (Systat Software Inc., San Jose, CA, USA). The principal component analysis (PCA) and Pearson’s correlation analysis were also carried out using the R language.

## 3 Results

### 3.1 Chalkiness, starch and protein content

**Figure 1:**
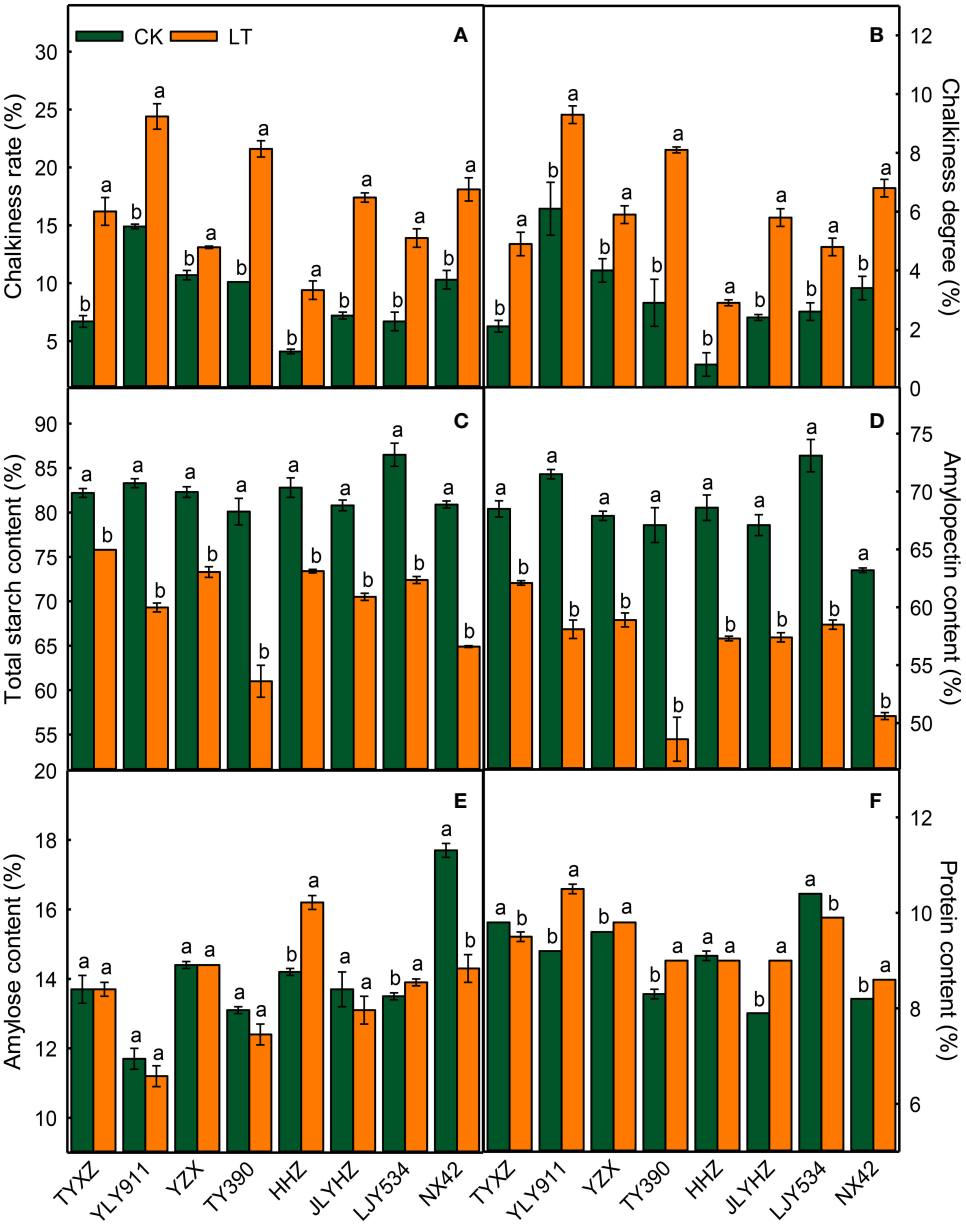
The impacts of low temperature on chalkiness rate **(A)**, chalkiness degree **(B)**, total starch content **(C)**, amylopectin content **(D)**, amylose content **(E)** and crude protein content **(F)** in high-quality indica rice. The values reported are mean ± standard error of three replicates. The different letters in each variety are statistically significant at p < 0.05. CK, control temperature treatment. LT, low-temperature treatment.

Notably, exposure to low temperatures during the grain-filling stage resulted in a significant increase in both the chalkiness rate and degree across all varieties (**Figures 1A, B**). The largest increases in chalkiness rate (11.5%) and chalkiness degree (5.2%) induced by lower temperature were seen in variety TY390. Compared to the control treatment, lower temperature resulted in a significant decrease in both total starch and amylopectin content across all rice varieties, with the greatest reduction observed in TY390 (19.1% and 18.5% for total starch and amylopectin, respectively) and the least in TYXZ (6.4% for both total starch and amylopectin) (**Figures 1C, D**). Conversely, the content of amylose decreased significantly only in NX42, while increased significantly in HHZ and LJY534 (**Figure 1E**). Notably, the effect of lower temperatures on protein content varied among rice varieties (**Figure 1F**), with a significant reduction observed in TYXZ by 3.1% and in LJY534 by 4.8%, and a significant increase observed in YLY911, YZX, TY390, JLYHZ, and NX42 by 2.1%-14.1%.

### 3.2 Thermal properties

The results of the study showed that variety, temperature, and their interactions had a significant effect on To, Tp, Tc, ΔH, R, and PHI, except for varietal effects on To (**Table 1**). YLY911, TY390, and JLYHZ exhibited higher gelatinization temperatures (To, Tp, and Tc) compared to other varieties. The ΔH of YZX, NX42, and HHZ was also higher than that of other varieties, while HHZ had the highest R among all varieties. When exposed to lower temperatures compared to the control, the To significantly increased in TYXZ, YLY911, and NX42, by 1.2% to 4.0%, whereas it notably decreased in YZX by 7.5% and TY390 by 0.7%. Under low temperature conditions, Tp increased significantly in TY390, TYXZ, JLYHZ, and NX42 (0.7%-1.8%), while Tc increased greatly only in TYXZ (0.8%) and decreased significantly in YLY911 (0.7%) and HHZ (1.6%). Low temperature caused a significant decrease in ΔH, R, and PHI for all varieties, except for the ΔH of TY390 and R of YZX, TY390, and JLYHZ. The largest decrease in ΔH was observed in HHZ (46.2%), followed by NX42 with 43.6%. LJY534 had the highest decrease in R with 11.5%, and the greatest decrease in PHI was observed in YZX by 49.6%.

**Table 1:** The impact of low temperature on the thermal properties of starch in high-quality indica rice.

Variety	Temperature treatment	To (°C)	Tp (°C)	Tc (°C)	ΔH (J g^-1^)	R (°C)	PHI
TYXZ	CK	67.2 ± 0.1 ^b^	74.8 ± 0.0 ^b^	78.0 ± 0.1 ^b^	3.76 ± 0.02 ^a^	10.75 ± 0.03 ^a^	0.49 ± 0.00 ^a^
	LT	69.9 ± 0.1 ^a^	75.6 ± 0.1 ^a^	78.6 ± 0.2 ^a^	2.66 ± 0.04 ^b^	8.66 ± 0.13 ^b^	0.47 ± 0.00 ^b^
YLY911	CK	75.6 ± 0.1 ^b^	82.0 ± 0.1 ^a^	85.5 ± 0.1 ^a^	4.14 ± 0.03 ^a^	9.93 ± 0.12 ^a^	0.64 ± 0.00 ^a^
	LT	76.7 ± 0.0 ^a^	82.1 ± 0.0 ^a^	84.9 ± 0.1 ^b^	2.98 ± 0.01 ^b^	8.21 ± 0.11 ^b^	0.55 ± 0.00 ^b^
YZX	CK	68.1 ± 0.2 ^a^	73.3 ± 0.2 ^a^	78.5 ± 0.5 ^a^	5.95 ± 0.01 ^a^	10.40 ± 0.28 ^b^	1.15 ± 0.01 ^a^
	LT	63.0 ± 0.1 ^b^	72.5 ± 0.2 ^a^	77.3 ± 0.1 ^a^	5.56 ± 0.05 ^b^	14.24 ± 0.17 ^a^	0.58 ± 0.00 ^b^
TY390	CK	77.0 ± 0.1 ^a^	82.9 ± 0.1 ^b^	86.3 ± 0.2 ^a^	3.66 ± 0.02 ^b^	9.28 ± 0.16 ^b^	0.63 ± 0.01 ^a^
	LT	76.5 ± 0.2 ^b^	83.5 ± 0.1 ^a^	86.8 ± 0.2 ^a^	3.75 ± 0.02 ^a^	10.28 ± 0.15 ^a^	0.54 ± 0.01 ^b^
HHZ	CK	67.5 ± 0.2 ^a^	75.1 ± 0.1 ^a^	79.8 ± 0.2 ^a^	5.45 ± 0.01 ^a^	12.27 ± 0.24 ^a^	0.73 ± 0.01 ^a^
	LT	67.5 ± 0.0 ^a^	75.3 ± 0.1 ^a^	78.5 ± 0.1 ^b^	2.93 ± 0.02 ^b^	11.01 ± 0.05 ^b^	0.38 ± 0.01 ^b^
JLYHZ	CK	75.8 ± 0.1 ^a^	81.4 ± 0.2 ^b^	85.7 ± 0.2 ^a^	4.09 ± 0.04 ^a^	9.81 ± 0.14 ^a^	0.73 ± 0.02 ^a^
	LT	76.7 ± 0.3 ^a^	82.9 ± 0.2 ^a^	86.1 ± 0.4 ^a^	2.83 ± 0.03 ^b^	9.37 ± 0.15 ^a^	0.46 ± 0.00 ^b^
LJY534	CK	67.3 ± 0.0 ^a^	74.5 ± 0.1 ^a^	77.9 ± 0.1 ^a^	3.65 ± 0.02 ^a^	10.61 ± 0.10 ^a^	0.51 ± 0.01 ^a^
	LT	67.9 ± 0.2 ^a^	74.1 ± 0.1 ^a^	77.3 ± 0.4 ^a^	2.53 ± 0.01 ^b^	9.39 ± 0.16 ^b^	0.41 ± 0.00 ^b^
NX42	CK	67.2 ± 0.1 ^b^	72.4 ± 0.0 ^b^	76.9 ± 0.1 ^a^	6.06 ± 0.01 ^a^	9.71 ± 0.05 ^a^	1.18 ± 0.01 ^a^
	LT	68.4 ± 0.1 ^a^	73.6 ± 0.0 ^a^	77.2 ± 0.1 ^a^	3.42 ± 0.02 ^b^	8.82 ± 0.07 ^b^	0.67 ± 0.01 ^b^
Analysis of variance							
Variety (V)		***	***	***	***	***	***
Temperature treatment (T)		ns	***	*	***	***	***
V*T		***	***	***	***	***	***

The values reported are mean ± standard error of three replicates. The different letters in the same column of each variety are statistically significant at p < 0.05. LSD (least significant difference) followed by *, ***, significance at 0.05 and 0.001, respectively, ns, non-significant. CK, control temperature treatment; LT, low-temperature treatment; To, onset temperature; Tp, peak temperature; Tc, conclusion temperature; ΔH, gelatinization enthalpy; R, gelatinization temperature range; PHI, peak height index.

### 3.3 Pasting properties

As it was shown in **Table 2**, variety, temperature, and their interactions had a significant effect on peak viscosity, trough viscosity, final viscosity, breakdown and setback. Compared to the control, exposure to low temperature resulted in a significant decrease in the peak viscosity, trough viscosity, and final viscosity of all varieties except for the trough viscosity in YZX and the final viscosity in HHZ. YLY911 and NX42 exhibited the largest reduction in peak viscosity and trough viscosity, with a decrease of 17.23% and 12.9%, respectively, whereas LJY534 and HHZ showed the least decrease, with reductions of 5.4% and 1.4%, respectively. JLYHZ showed the greatest decrease in final viscosity, by 9.3%, whereas a slight increase of 1.2% was observed in HHZ under low-temperature conditions. The decrease in breakdown induced by low temperature was significant in all varieties, with the greatest decrease observed in YLY911 by 25.6% and the least in LJY534 by 6.6%. However, the setback of all varieties increased significantly under low temperatures, with the greatest increase observed in HHZ by 262.4% and the least in YZX by 33.5%.

**Table 2:** The effects of low temperature on the pasting properties of high-quality indica rice.

Variety	Temperature treatment	Peak viscosity (cp)	Trough viscosity (cp)	Final viscosity (cp)	Breakdown (cp)	Setback (cp)
TYXZ	CK	3021.3± 6.3 ^a^	2039.3 ± 10.3 ^a^	2946.7 ± 8.2 ^a^	982.0 ± 4.0 ^a^	-74.7 ± 3.8 ^b^
	LT	2782.7± 9.4 ^b^	1906.7 ± 3.5 ^b^	2811.0 ± 8.5 ^b^	876.0 ± 7.0 ^b^	28.3 ± 0.9 ^a^
YLY911	CK	3627.0 ± 5.5 ^a^	2225.7 ± 6.2 ^a^	2976.0 ± 7.1 ^a^	1401.3 ± 10.8 ^a^	-651.0 ± 10.0 ^b^
	LT	3004.3 ± 19.2 ^b^	1962.0 ± 30.5 ^b^	2705.3 ± 2.3 ^b^	1042.3 ± 22.4 ^b^	-299.0 ± 17.2 ^a^
YZX	CK	3041.3 ± 3.8 ^a^	1851.7 ± 39.9 ^a^	2760.0 ± 15.0 ^a^	1189.7 ± 36.1 ^a^	-281.3 ± 11.4 ^b^
	LT	2844.7 ± 7.8 ^b^	1775.3 ± 7.9 ^a^	2657.7 ± 14.8 ^b^	1069.3 ± 14.9 ^b^	-187.0 ± 17.3 ^a^
TY390	CK	3426.0 ± 2.5 ^a^	1908.7 ± 3.3 ^a^	2742.3 ± 2.3 ^a^	1517.3 ± 2.6 ^a^	-683.7 ± 4.7 ^b^
	LT	3017.0 ± 13.1 ^b^	1780.7 ± 8.7 ^b^	2649.0 ± 9.6 ^b^	1236.3 ± 9.3 ^b^	-368.0 ± 3.5 ^a^
HHZ	CK	2965.7 ± 3.5 ^a^	1918.7 ± 3.9 ^a^	2850.7 ± 14.5 ^a^	1047.0 ± 1.5 ^a^	-115.0 ± 11.5 ^b^
	LT	2698.3 ± 6.2 ^b^	1891.7 ± 3.2 ^b^	2885.0 ± 11.0 ^a^	806.7 ± 7.0 ^b^	186.7 ± 12.9 ^a^
JLYHZ	CK	3312.0 ± 6.7 ^a^	2087.7 ± 19.9 ^a^	2972.7 ± 5.8 ^a^	1224.3 ± 18.9 ^a^	-339.3 ± 1.8 ^b^
	LT	2829.0 ± 7.5 ^b^	1837.0 ± 2.0 ^b^	2696.0 ± 4.2 ^b^	992.0 ± 9.3 ^b^	-133.0 ± 3.6 ^a^
LJY534	CK	2738.7 ± 17.9 ^a^	1927.7 ± 12.5 ^a^	2900.3 ± 8.7 ^a^	811.0 ± 5.5 ^a^	161.7 ± 9.3 ^b^
	LT	2590.7 ± 4.7 ^b^	1833.3 ± 5.0 ^b^	2835.3 ± 9.1 ^b^	757.3 ± 0.3 ^b^	244.7 ± 5.2 ^a^
NX42	CK	3475.7 ± 11.6 ^a^	2420.3 ± 11.5 ^a^	3345.3 ± 24.7 ^a^	1055.3 ± 1.5 ^a^	-130.3 ± 16.1 ^b^
	LT	2984.3 ± 11.7 ^b^	2109.3 ± 16.8 ^b^	3078.3 ± 9.2 ^b^	875.0 ± 5.3 ^b^	94.0 ± 2.6 ^a^
Analysis of variance						
Variety (V)		***	***	***	***	***
Temperature treatment (T)		***	***	***	***	***
V*T		***	***	***	***	***

The values reported are mean ± standard error of three replicates. The different letters in the same column of each variety are statistically significant at p < 0.05. LSD (least significant difference) followed by ***, significance at 0.001, CK, control temperature treatment. LT, low-temperature treatment.

### 3.4 Macro and micronutrients

**Figure 2:**
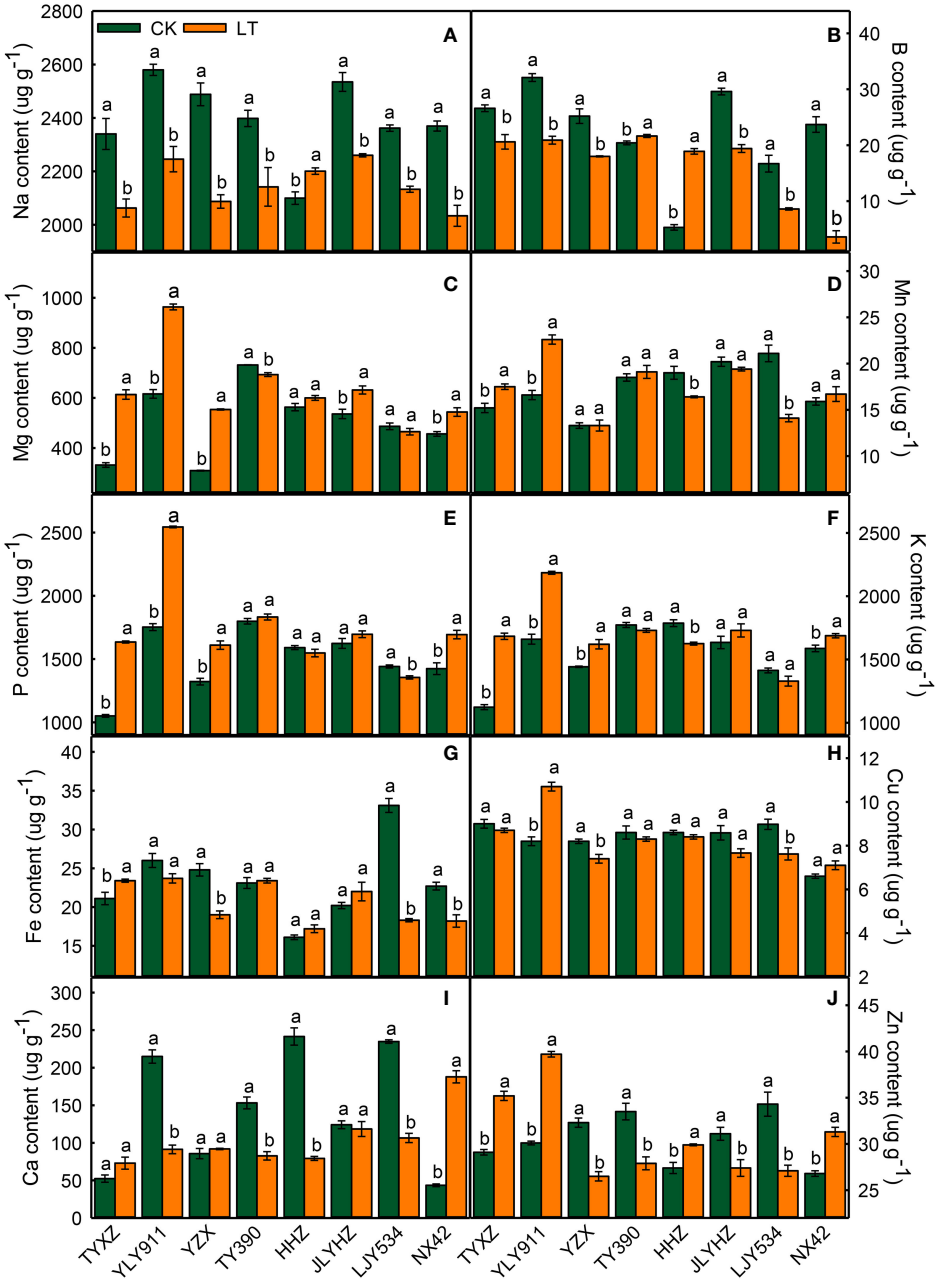
The impacts of low temperature on Na content **(A)**, B content **(B)**, Mg content **(C)**, Mn content **(D)**, P content **(E)**, K content **(F)**, Fe content **(G)**, Cu content **(H)**, Ca content **(I)** and Zn content **(J)** in high-quality indica rice. The values reported are mean ± standard error of three replicates. The different letters in each variety are statistically significant at p < 0.05. CK, control temperature treatment. LT, low-temperature treatment.

Mineral nutrient accumulation differed significantly across varieties, temperature treatments and their interactions. Exposure to lower temperature resulted in significant reductions of Na and B in all varieties, except for Na in HHZ, B in TY390 and HHZ (**Figures 2A, B**). Meanwhile, Mg levels were significantly increased in most of varieties except for TY390, HHZ and LJY534 (**Figure 2C**). When plants were exposed to low temperatures, Mn levels increased significantly in TYXZ and YLY911 but decreased greatly in HHZ and LJY534 (**Figure 2D**). P and K levels significantly increased in four varieties (TYXZ, YLY911, YZX and NX42), with noticeably decreases in LJY534 and HHZ, respectively (**Figures 2E, F**). Fe increased significantly only in TYXZ and decreased in YZX, LJY534 and NX42 (**Figure 2G**). The Cu levels changed slightly due to lower temperatures, increasing significantly only in YLY911 and decreasing greatly in YZX and LJY534 (**Figure 2H**). Among all varieties, significant decrease of Ca under low temperature were recorded in YLY911, TY390, HHZ and LJY534, but NX42 showed a noticeable increase in Ca (**Figure 2I**). Zn was affected by low temperatures in all varieties, with significant increases in TYXZ, YLY911, HHZ, and NX42, but decreased in remaining varieties (**Figure 2J**). YLY911 exhibited significant changes in nine elements, whereas TYXZ, YZX, NX42, and LJY534 had significant changes in eight elements.

### 3.5 Relationships among rice quality traits and varieties

**Figure 3:**
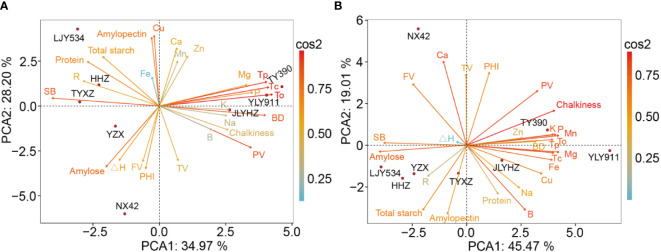
Principal component analysis of grain quality properties in high-quality indica rice under control **(A)** and low temperature **(B)** conditions. PV, peak viscosity; TV, trough viscosity; FV, final viscosity; BD, breakdown; SB, setback; To, onset temperature; Tp, peak temperature; Tc, tennination temperature; ΔH, gelatinization enthalpy; R, gelatinization temperature range; PHI, peak height index.

Principal component analysis (PCA) was conducted on all quality traits under both control and low-temperature conditions (**Figure 3**). The analysis revealed the tradeoffs and synergies among the quality traits of different varieties, which were classified into three distinct clusters for each temperature condition. The first cluster consisted of LJY534, HHZ, TYXZ, and YZX, which exhibited high protein and starch content as well as a high setback value. The second cluster comprised YLY911, TY390, and JLYHZ, which displayed high grain chalkiness, gelatinization temperature (To, Tp, Tc), breakdown, peak viscosity, and mineral nutrient content. The third cluster exclusively included NX42, which had high trough viscosity, final viscosity, and PHI. Furthermore, it was observed that the mineral nutrient content decreased in the first cluster and increased in the second cluster under low-temperature conditions.

**Figure 4:**
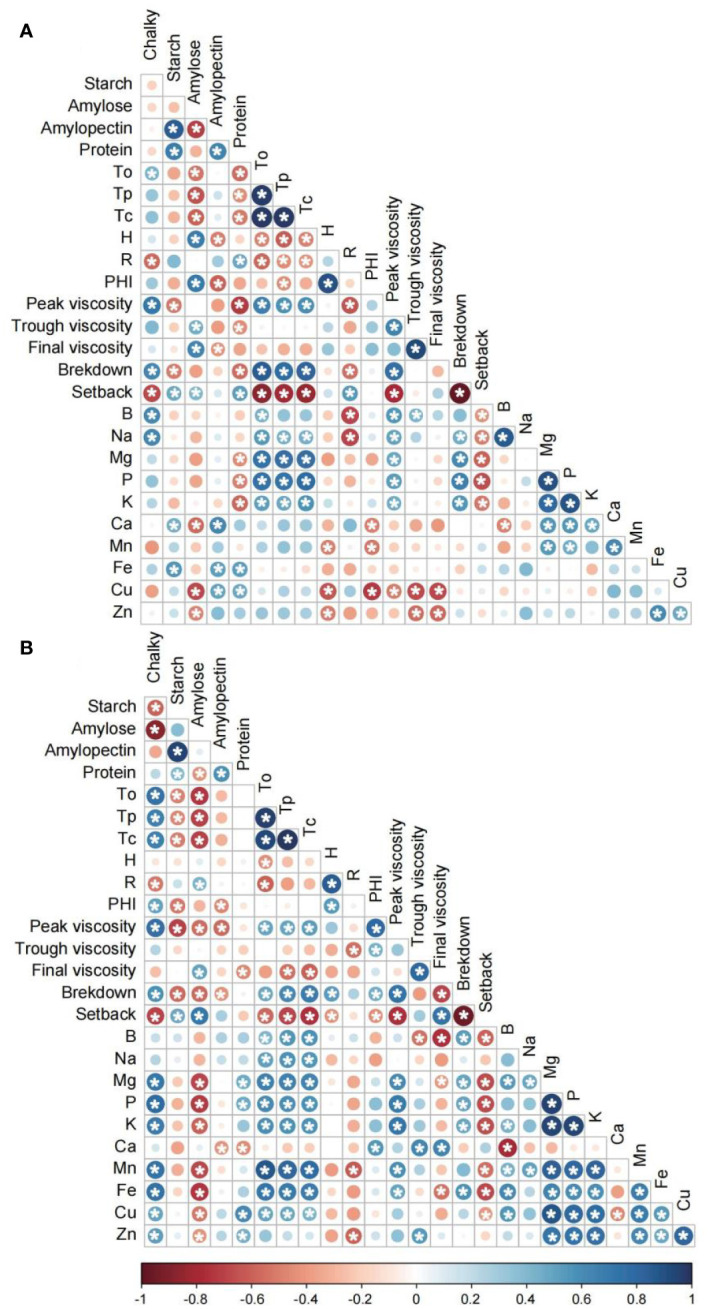
Correlation plot analysis of grain quality properties in high-quality indica rice under control **(A)** and low temperature **(B)** conditions. To, onset temperature; Tp, peak temperature; Tc, termination temperature;ΔH, gelatinization enthalpy; R, gelatinization temperature range; PHI, peak height index. P<0.05 followed by*.

The results of Pearson’s correlation analysis indicated significant relationships between various quality traits observed under two distinct temperature conditions (**Figure 4**). Notably, positive correlations were observed between grain chalkiness and To, peak viscosity, and breakdown, while negative correlations were observed with setback at the controlled temperature. Additionally, chalky was found to be negatively associated with starch and amylose content and positively associated with Tp, Tc and PHI under lower temperature conditions. The relationship between total starch or amylose content and To, Tp, Tc, peak viscosity, and breakdown was negative at the controlled temperature, with negative correlations becoming stronger at lower temperature. In contrast to total starch and amylose, protein content was found to be significantly correlated with thermal and pasting properties, including To, Tp, Tc, peak viscosity, trough viscosity, and breakdown, only at the controlled temperature, while the correlation was non-significant at lower temperature. Moreover, the present study identified noteworthy associations between quality traits and mineral nutrient contents in both controlled and low temperature conditions. Specifically, in the controlled condition, grain chalk exhibited a positive correlation with B and Na, while starch content showed a positive correlation with Ca and Fe. Conversely, amylose content was negatively related to Ca, Cu, and Zn. Notably, in contrast to control temperature, chalky grain, and amylose content demonstrated a greater number of positive and negative correlations with a range of mineral nutrient levels, including Mg, P, K, Mn, Fe, Cu, and Zn under low-temperature conditions. Minerals such as Na, Mg, P, and K were significantly correlated with To, Tp, Tc under both controlled and low temperature conditions, with the additional minerals B, Mn, Fe, and Cu showing positive associations with gelatinization temperature under low temperature conditions. Moreover, Mg, P, and K demonstrated positive correlations with peak viscosity and breakdown and negative correlations with setback under both controlled and low temperature conditions. In contrast, minerals Ca, Mn, and Fe were only significantly associated with pasting properties under low temperature conditions. Furthermore, significant positive correlations were observed among Mg, P, K, and Mn content under both temperature conditions. However, under low temperature conditions, Fe, Cu, and Zn exhibited a significant positive correlation with Mg, P, K, and Mn, which was not observed under controlled temperature conditions.

## 4 Discussion

### 4.1 Formation of grain chalkiness and changes in starch and protein in developing rice caryopses grown under low-temperature stress

Grain chalkiness is a notable visual trait identified as opaque white discoloration of the translucent endosperm, which is significantly affected by extreme environmental temperatures (Wada et al., 2019). Our results were in accordance with previous studies indicating lower temperature occurring during grain development triggers an increase in grain chalkiness (Zhang et al., 2019). Low-temperature stress during grain ripening facilitates the formation of chalky grains through the loose packing of arranged starch granules and the presence of large air spaces between them (Gong et al., 2013). However, few studies have shown either a decrease in chalkiness rate or no significant changes caused by low temperatures (Siddik et al., 2019; Lu et al., 2022). This discrepancy could be attributed to variations in the treatment temperature levels and growth stages at which the temperature treatment is applied. In the present study, the low temperature was applied during the second week of post-heading, which is considered a critical period for rice quality in response to extreme temperatures (Siddik et al., 2019).

The endosperm of rice is primarily composed of starch, followed by protein, and is susceptible to environmental changes (Wada et al., 2019). Our study supports previous research suggesting that low temperature during grain filling period can lead to a decrease in total starch accumulation (Zhu et al., 2017; Chen et al., 2022). This reduction is caused by a reduced activity of enzymes involved in starch synthesis, such as soluble starch synthase and starch branching enzyme (Chen et al., 2022), which results in lower levels of amylopectin and total starch contents. However, the effects on amylose content are inconsistent, with some studies reporting a decrease (Ai et al., 2023) and others an increase (Hu et al., 2020) in response to low temperature. In line with a previous study conducted on japonica rice (Zhu et al., 2017), two varieties demonstrated a significant increase in amylose under low temperatures while the other six varieties did not exhibit such an increase in this study. Lu et al. (2022) found that low temperature reduced amylose content in varieties with high amylose content but increased it in those with low amylose content, which discovered the impact of low temperature on amylose content varied depending on the initial amylose content of the rice varieties.

In this study, the protein content’s response to low temperatures exhibited variations, with both increases and decreases observed. These fluctuations can be attributed to varietal disparities, which have also been corroborated in prior research. Notably, some studies report a decline (Chun et al., 2015), while others indicate an increase (Zhang et al., 2019), and still, another group observes no significant change (Hu et al., 2020). Typically, rice with lower protein content tends to exhibit better cooking quality. In this study, TYXZ exhibited a notable decrease in protein content while showing the least reduction in total starch under low-temperature treatment. This observation suggests that TYXZ, among the eight varieties studied, might possess superior cold tolerance. Varietal differences in amino acid content may underlie these discrepancies, as amino acids can serve as signaling molecules that regulate resistance to abiotic stress (Ashraf and Foolad, 2007). Additionally, changes in protein content under abiotic stress could be associated with translocation from vegetative organs and amino acid synthesis in the rice grain (Kang et al., 2022). The variation in protein content under low temperature among rice varieties suggests genotypic differences, offering an opportunity for breeding more climate-resilient crops that can help address the new challenges to global health (Myers et al., 2014).

### 4.2 Thermal and pasting properties in response to low temperature during grain filling

Starch gelatinization is an endothermic process whereby starch crystallinity is lost in starch granules under specific heat and moisture conditions. Gelatinization temperature refers to the temperature peak at which rice absorbs water and starch granules swell irreversibly. The higher the gelatinization temperature of the grain, the firmer the core of cooked rice. The gelatinization temperature range (R) and gelatinization enthalpy △H are useful indicators of the energy required and cooking time necessary for starch gelatinization, and a higher △H and R require more energy to dissociate the helix structure of starch (Jiang et al., 2022). Therefore, the cooking quality of rice is primarily determined by its starch gelatinization properties. In our study, significant decreases in both gelatinization enthalpy △H, gelatinization temperature range R and PHI of starch under low temperatures were observed, which are consistent with previous research findings on japonica rice (Chun et al., 2015; Zhu et al., 2017). However, Ai et al. (2023) and Hu et al. (2020) found that the effect of low temperature on gelatinization enthalpy was not significant. These conflicting results may be attributed to the genetic diversity among rice, which was associated with factors such as amylose content, granular architecture, molecular weight distribution, and amylopectin fine structure (Kong et al., 2015). Previous studies also have indicated that the texture of cooked rice is associated with the fine structure of amylopectin, and that longer amylopectin chains forming double helices require higher temperatures to dissociate completely (Chun et al., 2015).

Rice with superior eating quality is characterized by higher breakdown and lower setback values, resulting in a soft texture after cooking and enhanced palatability. In this study, rice exposed to lower temperatures during grain filling exhibited a reduction in peak, trough, and final viscosities, a decline in breakdown, and an increase in setback, in comparison to rice exposed to the control temperature. These findings suggest that exposure to low-temperature stress during the grain filling period can negatively impact the eating quality of high-quality indica rice. Notably, these results align with previous research focused on japonica rice (Zhu et al., 2017; Hu et al., 2020). Moreover, the lower breakdown value and higher setback value observed under low-temperature conditions were associated with a decrease in crystallinity, a lower ratio of amylose to amylopectin, a decreased gelatinization enthalpy, and an increased size of starch granules (Hu et al., 2020; Ai et al., 2023).

### 4.3 Low temperatures during grain filling affect grain nutrient contents

Although rice is not known to be mineral-rich, it can still serve as an essential source of caloric energy and macro-micro nutrients for those who consume it as a staple food (Huang et al., 2020). Except for genotypic influences, environmental factors have been documented to affect minerals in rice grain (Du et al., 2013; Huang et al., 2016; Tiozon et al., 2023). Previous studies have shown that atmospheric CO_2_ levels and temperature (Myers et al., 2014; Chaturvedi et al., 2017), application of nitrogen (Gu et al., 2015; Wang et al., 2018) and water management (Xu et al., 2019) could affect the mineral nutrients of rice grains. This research did the first effort to explore the effects of low temperatures on macro and micro nutrients. Low temperatures significantly affected the mineral elements of brown rice in all varieties, although the extent of the impact varied among the different elements and varieties. The results are in line with previous studies that reported inconsistent changes in mineral content in rice grains to environments or managements (Chaturvedi et al., 2017). Our study showed that low temperatures during the grain filling stage decreased the content of Na and B, while increasing the levels of K, Mg and P among most of the varieties. The impact of low temperatures on Ca, Mn, Fe, Cu, and Zn content varied among the different rice varieties. Mineral elements in rice grains have been supplied by root uptake and translocation from vegetative plant tissues to developing rice grains during grain filling stage, and stomatal conductance and transpiration changing transpiration-driven mass flow of nutrients from root to apex organs (Houshmandfar et al., 2015). The present study provides a preliminary insight into the mineral accumulation in rice grains under low temperatures, while further investigation is needed to comprehensively understand the mineral uptake and distribution.

### 4.4 Relationships among rice quality traits were further enhanced under low temperatures

In this study, a negative correlation was observed between chalkiness and starch and amylose content when exposed to low temperatures. Additionally, Deng et al. (2021) reported a significant negative correlation between chalkiness and starch and amylose content under shading stress. Chalkiness is determined by the structure and arrangement of starch granules, where lower temperatures induce loosely packed, round starch granules in the chalk position due to defects in starch biosynthesis (Deng et al., 2018). The relationship between total starch or amylose content and gelatinization temperatures, peak viscosity, and breakdown was found to be negative at the controlled temperature, with the negative correlations becoming stronger at lower temperatures. Previous studies have also reported a negative correlation between amylose content and gelatinization temperature and breakdown, while a positive correlation with setback was observed (Zhong et al., 2022). However, the correlation between protein content and thermal and pasting properties was only significant at the controlled temperature, with no significant correlation at lower temperatures. Chun et al. (2015) reported a significant correlation between protein content, pasting viscosities, and setback value, with increasing treatment temperature. Furthermore, the palatability of cooked rice is influenced by the importance of starch components and protein content, indicating that amylopectin, amylose, and protein content are key chemical properties related to palatability (Chun et al., 2015). These findings suggest that protein content is associated with thermal and pasting properties, and its effect is amplified at higher temperatures. Moreover, the present study identified significant associations between quality traits and mineral nutrient contents under both controlled and low-temperature conditions. Xi et al. (2016) suggested that the mineral content of chalky grains was lower compared to translucent grains. However, our results align with Jiang et al. (2007) and Tiozon et al. (2023), who reported a significantly negative correlation between amylose content and K, Mg, and Mn levels. Additionally, significant positive correlations were observed among Mg, P, K, and Mn content under both temperature conditions, consistent with previous studies by Jiang et al. (2007) and Huang et al. (2016). Furthermore, the correlations between quality traits and mineral nutrients, as well as the relationships among mineral nutrient content, were more pronounced under low-temperature conditions than under controlled conditions. This finding is in line with Chaturvedi et al. (2017), who proposed that the associations between grain minerals and quality traits, such as protein and chalkiness, are further intensified under elevated CO_2_ levels combined with high temperature.

## 5 Conclusions

The results showed that low temperature decreased starch, amylopectin content, pasting viscosity, and breakdown, and increased chalkiness and setback. Gelatinization temperature and pasting properties showed stronger correlations with chalk, starch, and amylose content than with protein content under low temperatures. Notably, low temperature exerted a notable influence on the mineral nutrient contents, although the extent of this impact varied among different elements and rice varieties. Additionally, the mineral nutrient exhibited positive relationships with chalkiness, gelatinization temperature, peak viscosity, and breakdown, while negative associations were found with amylose content and setback. Furthermore, correlations among mineral nutrients and other quality traits were further amplified in the presence of low temperatures. The findings offer a basis for identifying candidate quality traits and their associations with mineral accumulation and cold tolerance in high-quality rice. This knowledge can be applied to develop stress-resistant rice varieties with improved nutritional quality.

## Data availability statement

The original contributions presented in the study are included in the article/**Supplementary Material**. Further inquiries can be directed to the corresponding authors.

## Author contributions

JY: Conceptualization, Data curation, Formal analysis, Writing – original draft, Writing – review & editing. XZ: Data curation, Formal analysis, Writing – original draft. DW: Investigation, Validation, Writing – original draft. JW: Investigation, Validation, Writing – original draft. HX: Investigation, Validation, Writing – original draft. YX: Investigation, Validation, Writing – original draft. HJX: Project administration, Resources, Writing – review & editing. WS: Funding acquisition, Project administration, Resources, Supervision, Writing – review & editing.
