# Supplementary figures and images for: The deterioration of starch physiochemical and minerals in high-quality indica rice under low-temperature stress during grain filling
Source: Front Plant Sci. 2024 Jan 22;14:1295003. doi: 10.3389/fpls.2023.1295003 (PMC10839034; doi:10.3389/fpls.2023.1295003)

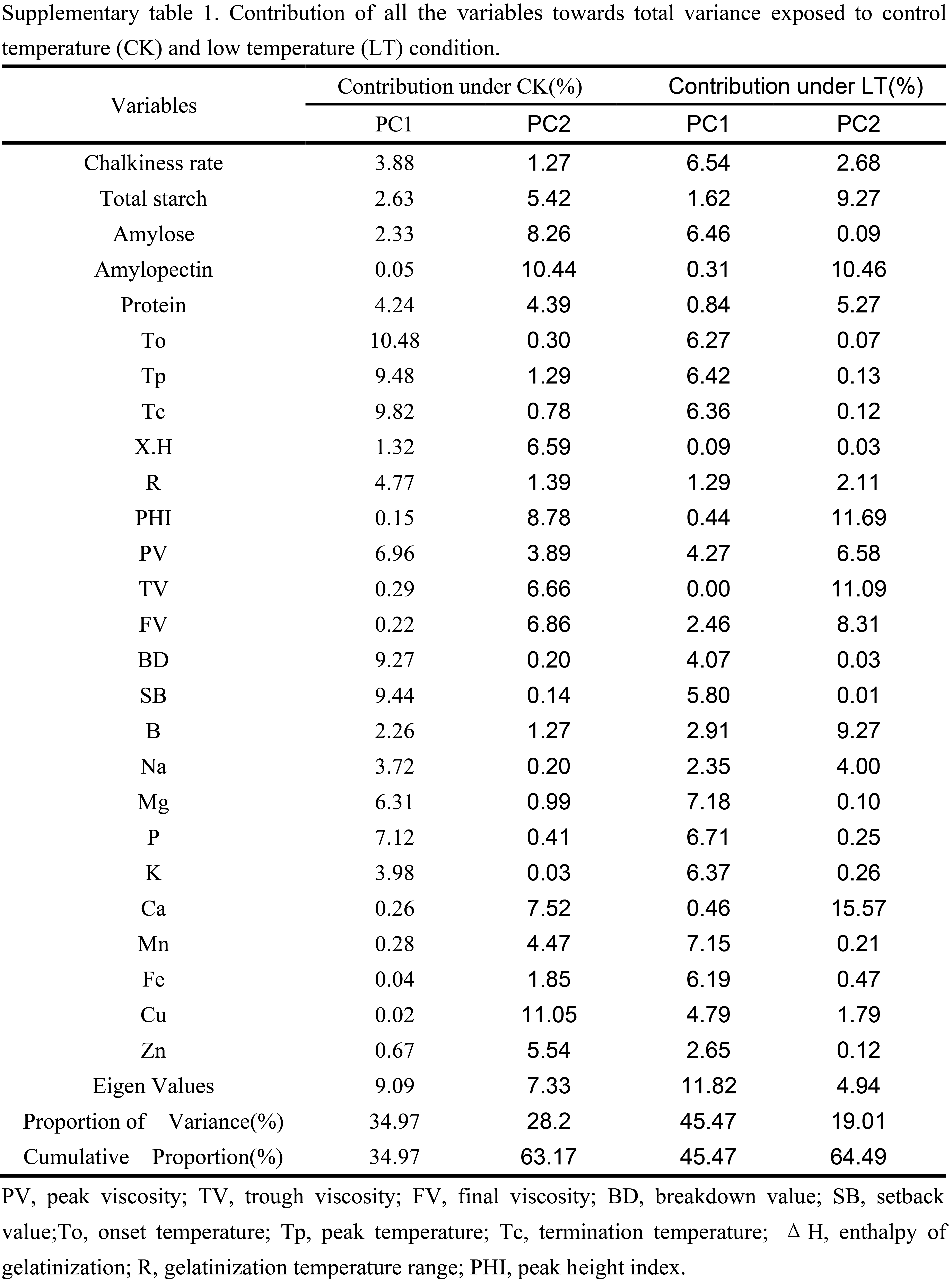

Supplement: Supplementary file 1 [file Image_1.jpeg]
